# Multidisciplinary Management of Failed Endodontic Treatment in a Hypertensive Patient With Severe Dental Anxiety Using Implant-Supported Rehabilitation: A Case Report

**DOI:** 10.7759/cureus.112619

**Published:** 2026-07-13

**Authors:** Keily J Portillo Avila

**Affiliations:** 1 Dentistry, Dental Loft, San Pedro Sula, HND

**Keywords:** dental anxiety, digital implant planning, failed endodontic treatment, hypertension, implant-supported rehabilitation, interdisciplinary care, oral surgery, patient-centered care, root canal failure, shared decision-making

## Abstract

Failed endodontic treatment may result in persistent pain and tooth loss, requiring careful evaluation to determine the most appropriate treatment approach. In medically compromised patients with severe dental anxiety, successful management also depends on medical stabilization, individualized treatment planning, and interdisciplinary collaboration.

A 56-year-old female with a medical history significant for hypertension and severe dental anxiety presented with intense pain associated with a previously endodontically treated mandibular left first molar (tooth #19). The patient's baseline blood pressure was 160/80 mmHg; however, during the initial dental evaluation, it increased to 180/140 mmHg, prompting postponement of treatment and immediate communication with her primary care physician. The patient was referred for medical stabilization before definitive dental care was undertaken. After medical clearance, comprehensive clinical and radiographic examinations, including cone beam computed tomography (CBCT), confirmed failed endodontic treatment with an unfavorable prognosis. Following discussion of all treatment options and shared decision-making, the patient elected extraction followed by delayed implant-supported rehabilitation. Atraumatic tooth extraction and alveolar ridge preservation were performed using a deproteinized bovine bone mineral xenograft covered with a resorbable collagen membrane. A delayed two-stage implant protocol was selected to allow maturation of the grafted site before implant placement. Three months later, CBCT-guided digital implant planning was used to determine optimal implant dimensions, and a 5 × 13 mm dental implant was successfully placed. Following successful osseointegration, the restorative phase was completed using a digital workflow with a scan body and a definitive implant-supported crown. During rehabilitation, malocclusion and dental crowding were identified, resulting in referral for interdisciplinary orthodontic treatment. At the one-year follow-up, the patient demonstrated successful implant function, favorable peri-implant tissue health, restored masticatory function, and high satisfaction with the overall treatment. This case highlights the importance of comprehensive medical assessment, physician consultation, patient-centered decision-making, delayed implant placement following ridge preservation, digital implant planning, and interdisciplinary collaboration in achieving safe and predictable outcomes in medically compromised patients presenting with severe dental anxiety and failed endodontic treatment.

## Introduction

Dental anxiety is one of the most common barriers to seeking oral healthcare and affects millions of individuals worldwide [1-5]. Patients with moderate to severe dental anxiety frequently delay or avoid routine dental treatment, increasing their risk of advanced oral disease, persistent pain, odontogenic infections, and tooth loss. In addition to its impact on oral health, dental anxiety activates the sympathetic nervous system, resulting in transient elevations in blood pressure, heart rate, and circulating stress hormones. These physiologic responses are particularly important in patients with preexisting hypertension because they may increase the risk of adverse cardiovascular events during dental treatment.

The management of hypertensive patients in the dental setting requires careful medical assessment, appropriate monitoring of vital signs, effective anxiety reduction strategies, and close communication with the patient's primary care physician when blood pressure is inadequately controlled or acute hypertensive episodes are suspected [6,7]. Current clinical recommendations emphasize postponing elective invasive dental procedures in patients with significantly elevated blood pressure until adequate medical stabilization has been achieved. This patient-centered approach prioritizes patient safety while allowing definitive dental treatment to proceed under more favorable medical conditions.

Persistent odontogenic infections resulting from failed endodontic treatment remain a common indication for tooth extraction when nonsurgical retreatment or endodontic surgery is unlikely to provide a predictable long-term outcome [8,9]. Treatment decisions should be based on a comprehensive clinical and radiographic evaluation, remaining tooth structure, periodontal status, restorability, patient preferences, and overall prognosis. When extraction is indicated, implant-supported rehabilitation represents a predictable treatment option capable of restoring oral function, esthetics, and long-term stability [10-12].

Recent advances in digital dentistry have further enhanced implant therapy through the integration of cone beam computed tomography (CBCT), three-dimensional treatment planning, guided implant positioning, and digital restorative workflows [13-14]. These technologies enable accurate assessment of bone volume, prosthetically driven implant placement, and precise fabrication of implant-supported restorations using intraoral digital impressions and scan bodies, contributing to improved surgical and restorative accuracy.

This case report describes the multidisciplinary management of a hypertensive patient with severe dental anxiety and a persistent odontogenic infection secondary to failed endodontic treatment. The coordinated care pathway included physician consultation and medical stabilization, patient-centered shared decision-making, atraumatic tooth extraction with alveolar ridge preservation, CBCT-guided digital implant planning, implant-supported rehabilitation using a digital restorative workflow, and subsequent interdisciplinary orthodontic referral. This comprehensive approach resulted in favorable clinical, functional, and esthetic outcomes and highlights the importance of multidisciplinary collaboration in the management of medically compromised patients requiring complex oral rehabilitation [15-19].

## Case presentation

A 56-year-old female presented with severe pain associated with the mandibular left first molar (tooth #19). Her medical history was significant for hypertension managed by her primary care physician and severe dental anxiety. The patient reported that several dental providers had previously declined treatment because her blood pressure consistently increased upon entering the dental office, preventing definitive management of her chief complaint. She described persistent pain that interfered with mastication and significantly affected her quality of life.

During the initial visit, the patient exhibited marked anxiety upon entering the dental clinic. Her baseline blood pressure, as documented by her primary care physician, was 160/80 mmHg. Upon arrival at the dental office, her blood pressure increased to 180/140 mmHg. Despite efforts to reassure and calm the patient, the blood pressure remained markedly elevated. Considering the potential cardiovascular risk associated with proceeding under these conditions, definitive dental treatment was postponed. The treating dentist contacted the patient's primary care physician and referred the patient for medical evaluation and stabilization before any invasive dental procedure was performed. Following medical assessment and appropriate blood pressure control, the patient was medically cleared to proceed with definitive dental treatment.

A comprehensive clinical and radiographic examination was subsequently performed. Intraoral examination revealed a previously endodontically treated mandibular left first molar restored with a full coverage crown. Persistent pain and radiographic evidence of post-treatment apical periodontitis indicated endodontic treatment failure and an unfavorable long-term prognosis (Figure 1). After discussing all available treatment options, including nonsurgical endodontic retreatment, endodontic surgery, extraction, and implant-supported rehabilitation, the patient elected extraction followed by implant-supported rehabilitation through a shared decision-making process.

**Figure 1:**
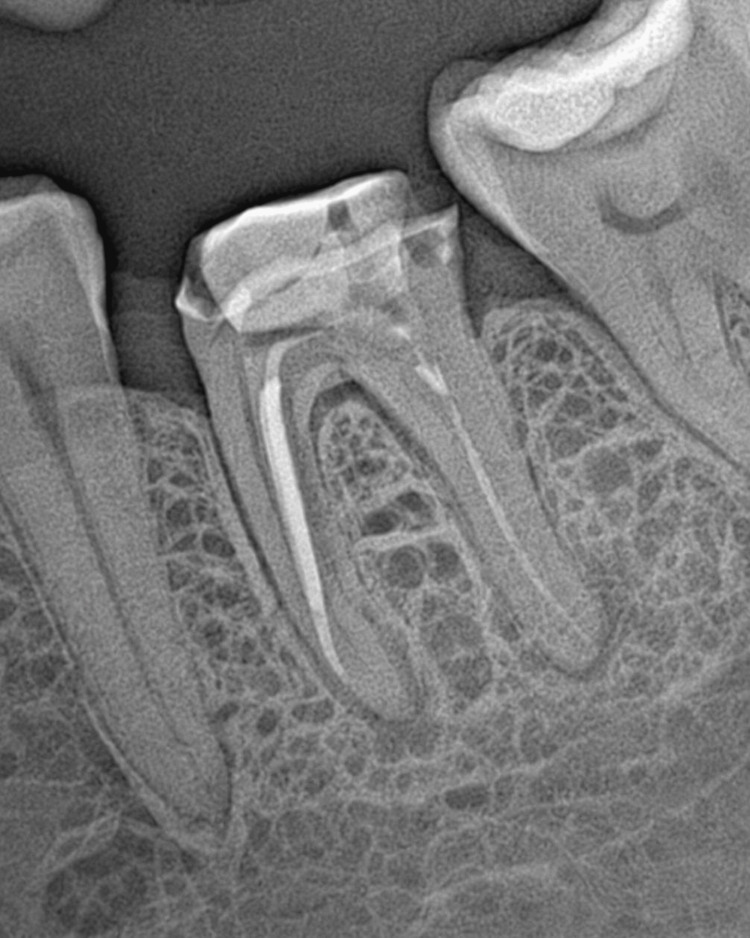
Initial Periapical Radiograph of Tooth #19. Periapical radiograph demonstrating a previously endodontically treated mandibular left first molar (tooth #19) with persistent periapical radiolucency consistent with post-treatment apical periodontitis. The radiographic findings, together with the patient's clinical presentation, indicated endodontic treatment failure and an unfavorable long-term prognosis, supporting extraction followed by implant-supported rehabilitation.

The tooth was atraumatically extracted under local anesthesia, and alveolar ridge preservation was performed using a particulate deproteinized bovine bone mineral xenograft covered with a resorbable collagen membrane to preserve ridge dimensions for future implant placement (Figure 2). Postoperatively, prophylactic antibiotic therapy was prescribed together with appropriate analgesics and postoperative instructions. Because alveolar ridge preservation with bone grafting was performed, a delayed two-stage implant protocol was selected rather than immediate implant placement. This approach allowed adequate healing and maturation of the grafted site before implant placement, optimizing bone quality and volume for prosthetically driven implant positioning, predictable primary stability, and successful osseointegration. Healing progressed uneventfully. Clinical follow-up demonstrated satisfactory early postoperative healing, and after approximately three months, complete soft tissue maturation and preservation of the alveolar ridge architecture were observed, confirming adequate healing for implant placement (Figure 3).

**Figure 2:**
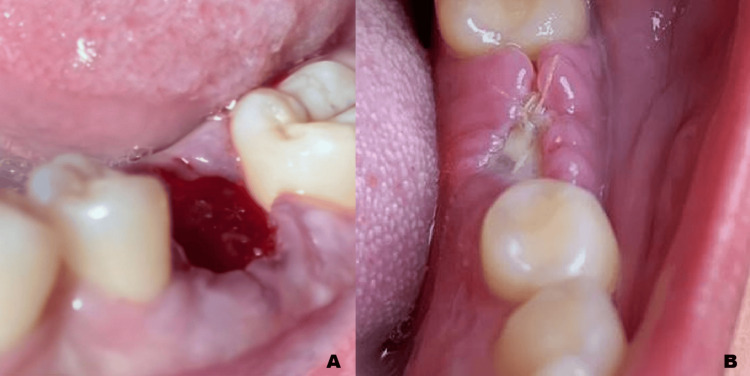
Atraumatic Tooth Extraction and Alveolar Ridge Preservation. (A) Clinical photograph obtained immediately following atraumatic extraction of tooth #19, demonstrating placement of particulate bone graft material within the extraction socket and coverage with a resorbable collagen membrane for alveolar ridge preservation. (B) Clinical photograph obtained during the early postoperative healing phase, demonstrating satisfactory initial soft tissue healing with stabilization of the grafted site.

**Figure 3:**
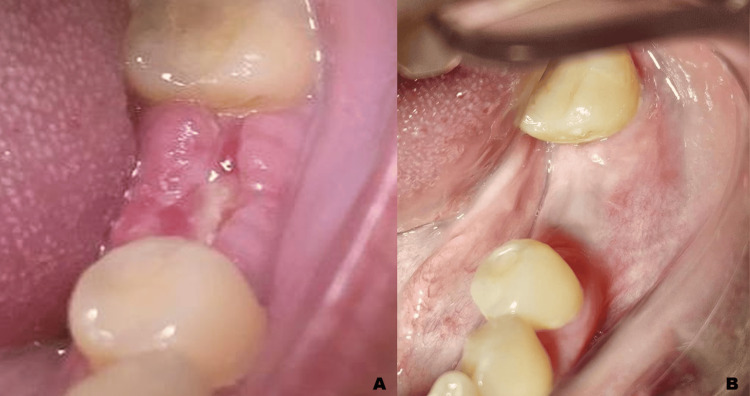
Soft Tissue Healing Following Tooth Extraction and Alveolar Ridge Preservation. (A) Immediate postoperative clinical view following atraumatic tooth extraction and alveolar ridge preservation, showing the fresh extraction socket with particulate bone graft material covered by a resorbable collagen membrane. (B) Clinical photograph obtained three months after atraumatic tooth extraction and alveolar ridge preservation, demonstrating complete soft tissue maturation and preservation of the alveolar ridge architecture, with adequate healing for implant placement.

CBCT was then obtained for prosthetically driven digital implant planning. Three-dimensional analysis demonstrated adequate bone volume and allowed determination of the optimal implant position, angulation, and dimensions (Figure 4). A 5 × 13 mm MIS conical dental implant was placed with satisfactory primary stability, and the immediate postoperative periapical radiograph confirmed appropriate implant positioning. Following an uneventful osseointegration period, second-stage surgery (implant uncovering) was performed, and a healing abutment was connected to allow peri-implant soft tissue maturation before the restorative phase (Figure 5).

**Figure 4:**
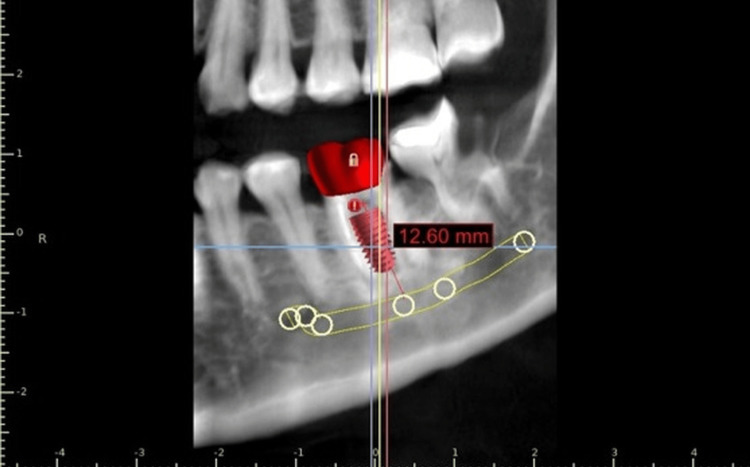
Cone Beam Computed Tomography (CBCT) Guided Digital Implant Planning. Three-dimensional CBCT evaluation of the edentulous site following three months of healing after tooth #19 extraction and alveolar ridge preservation. Digital implant planning was performed to assess the available bone volume, determine the optimal implant position, angulation, and dimensions, and facilitate prosthetically driven placement of a 5 × 13 mm MIS conical dental implant, ensuring appropriate anatomical positioning and restorative outcomes.

**Figure 5:**
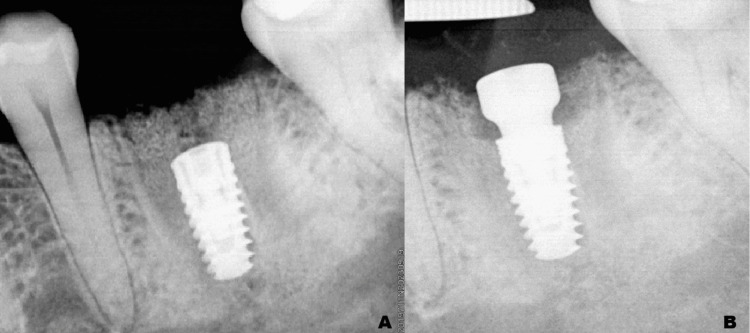
Radiographic Evaluation Following Implant Placement and Second Stage Surgery. (A) Immediate postoperative periapical radiograph obtained following placement of a 5 × 13 mm MIS conical dental implant after three months of alveolar ridge healing. The implant demonstrated appropriate three-dimensional positioning within the regenerated ridge, favorable angulation, and satisfactory primary stability. (B) Periapical radiograph obtained immediately after second-stage surgery (implant uncovering), demonstrating successful osseointegration of the previously submerged 5 × 13 mm MIS conical dental implant. A healing abutment was connected to facilitate peri-implant soft tissue maturation before definitive prosthetic rehabilitation. The surgical flap was repositioned and secured with sutures.

After adequate soft tissue maturation, the healing abutment was removed, revealing a healthy peri-implant soft tissue profile. A scan body was connected, and a digital intraoral impression was obtained and transmitted to the dental laboratory for fabrication of the definitive screw-retained implant-supported crown (Figure 6). The definitive restoration demonstrated satisfactory marginal adaptation, occlusion, esthetics, and function. Clinical and radiographic examinations confirmed successful implant rehabilitation with stable peri-implant bone levels (Figure 7).

**Figure 6:**
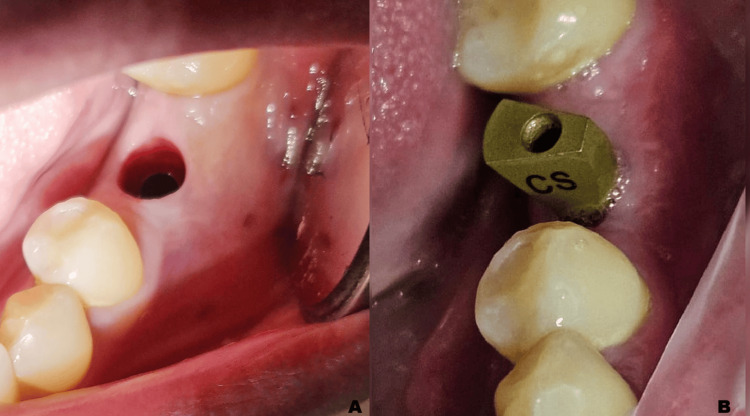
Digital Impression Workflow for Definitive Implant Restoration. (A) Clinical photograph demonstrating removal of the healing abutment after successful peri-implant soft tissue maturation. The peri-implant tissues exhibited healthy healing and a well-developed emergence profile, providing optimal conditions for placement of the scan body and acquisition of a digital intraoral impression. (B) Clinical photograph demonstrating placement of the scan body onto the osseointegrated implant following removal of the healing abutment. The stable scan body enabled acquisition of a digital intraoral impression, which was transmitted to the dental laboratory for fabrication of the definitive screw-retained implant-supported crown.

**Figure 7:**
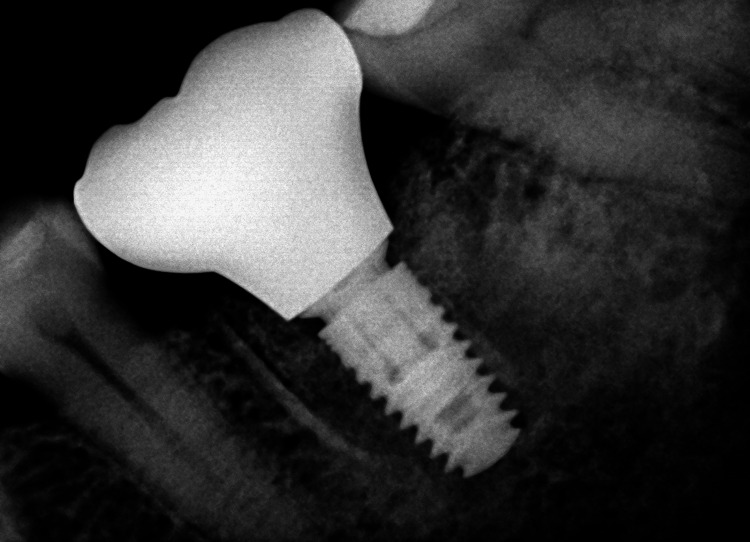
Definitive Implant-Supported Crown. Periapical radiograph demonstrating the definitive screw-retained implant-supported crown in function. The restoration exhibits appropriate implant positioning, complete osseointegration, stable peri-implant crestal bone levels, and satisfactory prosthetic adaptation, indicating successful functional and radiographic rehabilitation.

During the restorative phase, generalized dental crowding and malocclusion were identified that could compromise long-term occlusal stability. Consequently, the patient underwent interdisciplinary orthodontic treatment following implant rehabilitation. At the one-year follow-up, the patient remained asymptomatic, with healthy peri-implant soft tissues, stable radiographic bone levels, satisfactory implant function, restoration of comfortable mastication, and excellent esthetic and functional outcomes. Completion of orthodontic therapy resulted in harmonious integration of the implant-supported restoration within the dental arch, demonstrating the value of a multidisciplinary treatment approach (Figure 8).

**Figure 8:**
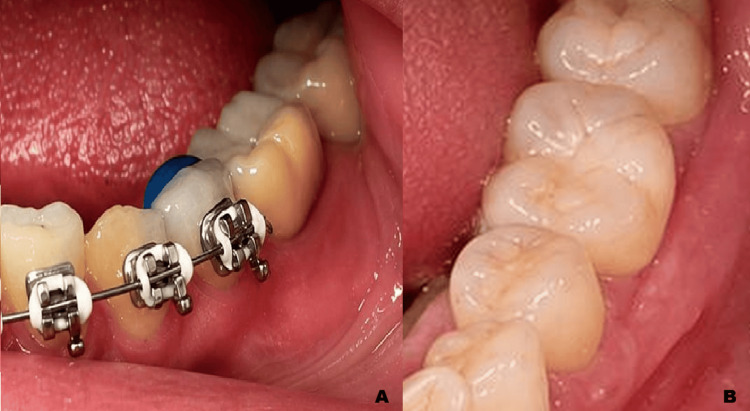
Interdisciplinary Management Following Implant Rehabilitation. (A) Clinical photograph demonstrating the definitive implant-supported crown in function during comprehensive orthodontic treatment. The patient underwent orthodontic therapy as part of an interdisciplinary treatment approach to improve dental alignment, correct the existing malocclusion, and optimize functional and esthetic outcomes. (B) Clinical photograph demonstrating the final interdisciplinary treatment outcome, with the definitive implant-supported crown exhibiting healthy peri-implant soft tissues, satisfactory alignment with the adjacent dentition, and harmonious integration into the dental arch following completion of orthodontic treatment.

## Discussion

Dental anxiety remains one of the most prevalent barriers to oral healthcare and is associated with delayed treatment, avoidance of dental visits, and progression of oral disease. Activation of the sympathetic nervous system during periods of intense anxiety results in increased catecholamine release, producing transient elevations in blood pressure and heart rate. These physiologic responses may be particularly pronounced in patients with preexisting hypertension and may increase the risk of adverse cardiovascular events during dental procedures. Consequently, appropriate anxiety management, continuous monitoring of vital signs, effective communication, and individualized treatment planning are essential components of safe dental practice [1-5].

The successful management of medically compromised patients in dentistry requires careful integration of medical assessment, patient-centered communication, evidence-based decision-making, and interdisciplinary collaboration. This case illustrates how severe dental anxiety, uncontrolled blood pressure at presentation, and chronic odontogenic infection may complicate routine dental care and emphasizes the importance of prioritizing patient safety before definitive treatment. Rather than proceeding with an invasive procedure during an episode of markedly elevated blood pressure, treatment was postponed until medical stabilization was achieved through consultation with the patient's primary care physician. This approach is consistent with current recommendations advocating medical evaluation before elective dental treatment in patients presenting with significantly elevated blood pressure or suspected cardiovascular instability [6,7].

Management of failed endodontically treated teeth continues to represent a common clinical challenge; however, treatment decisions should remain individualized and based on a comprehensive evaluation of the clinical findings, restorability, periodontal support, remaining tooth structure, patient preferences, and long-term prognosis. Although nonsurgical endodontic retreatment and endodontic microsurgery remain appropriate treatment options in selected cases, extraction followed by implant-supported rehabilitation may provide a predictable solution when the prognosis of the natural tooth is unfavorable. In the present case, shared decision-making allowed the patient to actively participate in treatment selection after receiving a comprehensive explanation of all available therapeutic options, reinforcing the principles of patient autonomy and informed consent [8,9].

Following extraction, alveolar ridge preservation using a deproteinized bovine bone mineral xenograft and a resorbable collagen membrane helped maintain ridge dimensions and facilitated a delayed two-stage implant protocol. Delaying implant placement for approximately three months allowed adequate healing and maturation of the grafted site before implant insertion, optimizing bone quality and volume, facilitating prosthetically driven implant positioning, and contributing to predictable primary stability and successful osseointegration. Contemporary evidence demonstrates that ridge preservation reduces post-extraction dimensional changes and may decrease the need for additional regenerative procedures before implant placement [10-12].

Digital technologies have significantly improved the predictability of implant therapy. The use of CBCT enabled three-dimensional assessment of the recipient site, facilitating prosthetically driven implant planning and accurate selection of implant dimensions. Careful evaluation of the regenerated bone volume and implant positioning contributed to safe implant placement while minimizing surgical risk [13,14].

Following successful osseointegration, the restorative phase was completed using a fully digital workflow incorporating a scan body for implant-level digital impression acquisition. Digital restorative techniques have improved communication between clinicians and dental laboratories while enhancing the precision of implant-supported prosthetic fabrication, emergence profile development, and occlusal accuracy [15,16]. Systematic reviews have demonstrated excellent survival, success, and long-term clinical performance of implant-supported single crowns, supporting their predictability as a treatment option for single-tooth rehabilitation [17,18].

An additional educational aspect of this case is the value of interdisciplinary care. Successful management extended beyond extraction and implant rehabilitation to include medical consultation before surgery and orthodontic referral following definitive restoration. A comprehensive orthodontic evaluation is essential for identifying occlusal discrepancies, periodontal considerations, and factors that may influence long-term treatment stability and oral health. Coordinated interdisciplinary treatment planning allows clinicians to optimize functional and esthetic outcomes while preserving periodontal health and improving the overall quality of patient care [19].

Although this report describes a single patient and therefore cannot establish causality or be generalized to all clinical situations, it demonstrates the practical application of evidence-based principles in the management of a medically compromised patient. Larger prospective clinical studies are warranted to further investigate the relationship between severe dental anxiety, transient hypertensive responses, chronic odontogenic infection, delayed implant rehabilitation following ridge preservation, and long-term implant outcomes. Nevertheless, this case provides a clinically relevant example of how evidence-based clinical decision-making, individualized treatment planning, digital technologies, and interdisciplinary collaboration can contribute to safe, predictable, and successful outcomes in medically compromised patients requiring complex oral rehabilitation.

## Conclusions

This case demonstrates that successful management of medically compromised patients extends beyond the technical aspects of dental treatment and requires careful medical assessment, patient-centered communication, evidence-based clinical decision-making, and interdisciplinary collaboration. Appropriate physician consultation and medical stabilization before invasive treatment allowed safe management of a hypertensive patient with severe dental anxiety and failed endodontic treatment, ultimately resulting in successful implant-supported rehabilitation and restoration of oral function. The integration of CBCT-guided digital implant planning, a digital restorative workflow, shared decision-making, and coordinated orthodontic referral was associated with favorable clinical and functional outcomes in this case. The comprehensive multidisciplinary management strategy described in this report may provide a useful educational example for clinicians managing similar patients. While the findings from a single case cannot be generalized, the systematic approach presented may assist clinicians when considering similar management strategies for carefully selected patients by emphasizing patient safety, individualized clinical judgment, and interdisciplinary collaboration.
